# Sodium-glucose co-transporter 2 inhibitors are negatively correlated with hypomagnesemia in elderly patients with type 2 diabetes

**DOI:** 10.3389/fnut.2026.1790168

**Published:** 2026-05-14

**Authors:** Leilei Lin, Yi Jin, Lanling Jin, Binfeng Wang, Jie Xu, Senzhong Chen

**Affiliations:** 1 Department of Gerontology, Wenzhou Central Hospital, Affiliated to Wenzhou Medical University, Wenzhou, Zhejiang, China; 2 Department of Rheumatology, The Second Affiliated Hospital of Wenzhou Medical University, Wenzhou, Zhejiang, China; 3 Department of Neurology, Pujiang County People’s Hospital, Wenzhou, Jinhua, Zhejiang, China; 4 Department of Gastroenterology, Yueqing Hospital Affiliated to Wenzhou Medical University, Wenzhou, Zhejiang, China

**Keywords:** aged, hypomagnesemia, SGLT2I, sodium-glucose co-transporter 2 inhibitor, T2DM

## Abstract

**Introduction:**

The rapidly aging demographic is contributing to a significant increase in diabetes prevalence, particularly Type 2, which subsequently elevates the risk of hypo-magnesemia. Polypharmacy is recognized as a risk factor for hypomagnesemia, while the association between SGLT2 inhibitors (SGLT2i) and hypomagnesemia remains underreported.

**Methods:**

To investigate this relationship, a retrospective cross-sectional study was conducted on patients aged 60 and older with Type 2 dia-betes, utilizing data from Wenzhou Central Hospital between January 2023 and December 2024. Multivariable logistic regression and propensity score match-ing (PSM) analyses were employed to compute adjusted odds ratios (ORs) for hypomagnesemia, accounting for potential confounders.

**Results:**

The study enrolled 2,827 subjects with a mean age of 72.59 years, of which 485 (17.2%) were diag-nosed with hypomagnesemia. Both univariate [OR 0.481, 95% CI (0.334–0.692), *p* < 0.001] and multivariate [OR 0.392, 95% CI (0.267–0.576), *p* = 0.002] analyses revealed a negative correlation between SGLT2i use and hypomagnesemia. A similar trend was observed in the PSM analysis, with univariate [OR 0.353, 95% CI (0.230–0.540), *p* < 0.001] and multivariate [OR 0.325, 95% CI (0.208–0.506), *p* < 0.001] results. Furthermore, elevated levels of HbA1c and the administra-tion of metformin were associated with a higher incidence of hypomagnesemia, corroborating both pre-PSM and post-PSM analyses.

**Discussion:**

Our findings suggest that among elderly patients, it may be prudent to consider serum mag-nesium levels when determining the appropriate glucose—lowering medication to initiate. SGLT2i is negatively correlated with hypomagnesemia.

## Introduction

Type 2 diabetes is characterized by cellular and extracellular magnesium depletion (1). Hypomagnesemia is approximately tenfold more prevalent in individuals with type 2 diabetes (T2D) compared to the general population (2). It is associated with insulin resistance, hyperlipidemia, hyperglycemia, and accelerated progression of the disease (3–5). To sustain appropriate glucose levels and blood pressure, polypharmacy becomes an inescapable aspect of effectively managing type 2 diabetes (6). Nonetheless, it concurrently emerges as an escalating risk factor. Previous studies have consistently found that the use of various antidiabetic medications, particularly metformin, sulfonylurea derivatives, and DPP4 inhibitors, increases the incidence of hypomagnesemia (7–9). Although no cohort studies have specifically addressed the relationship between SGLT2 inhibitors and hypomagnesemia, meta-analyses have suggested that SGLT2 inhibitors slightly elevate serum magnesium levels in patients with type 2 diabetes (10–12).

The World Health Organization (WHO) defines older individuals as those aged 60 years or older (13, 14). There is a robust correlation between advancing age and the prevalence of Type 2 Diabetes Mellitus (T2DM), with older adults, now representing nearly half of all adults diagnosed with diabetes mellitus (15, 16). Previous studies have suggested that magnesium intake and its absorption by the gastrointestinal tract tend to diminish with advancing age (17, 18). Despite the elderly population with T2DM being a high-risk group for hypomagnesemia, there has been limited focus on this demographic in past studies. Furthermore, after the establishment of glycemic control regimens, older individuals frequently become lost to follow-up in outpatient settings and often continue with a static treatment plan, underscoring the necessity for a comprehensive evaluation of potential adverse drug reactions.

## Methods

### Study population

**Figure 1:**
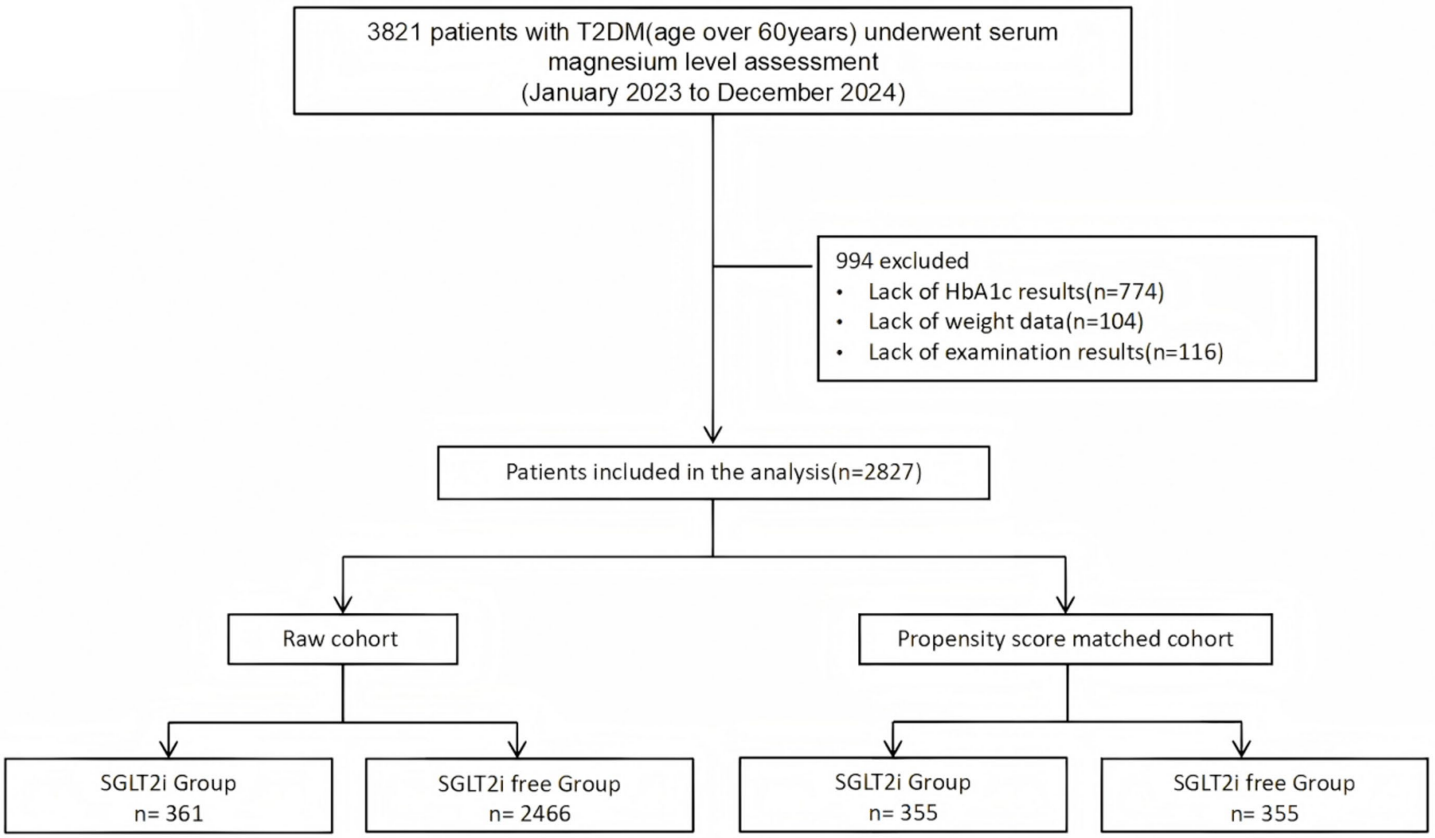
Study flowchart to illustrate the study selection. T2DM, type 2 diabetes mellitus; SGLT2i, sodium-glucose co-transporter 2 inhibitor.

We conducted a retrospective cross-sectional study at Wenzhou Central Hospital between January 2023 to December 2024. This research was approved by the ethics committee of Wenzhou Central Hospital and adhered strictly to the Declaration of Helsinki (approval number: L2024-12-022). Due to the retrospective nature of the study, Ethics Committee of Wenzhou Central Hospital waived the need of obtaining informed consent. We included an elderly population aged 60 years or older with type 2 diabetes who underwent serum magnesium level assessment. However, individuals with a history of malignancy, those with connective tissue diseases, and those currently taking diuretics were excluded from the study. The specific screening process is presented in Figure 1.

### SGLT2i exposure

According to the exposure of SGLT2i, patients were divided into the SGLT2i free group and the SGLT2i group. The SGLT2i group consisted of patients receiving SGLT2i more than 3 month. Individuals classified as SGLT2i-free users are defined as those who have not used SGLT2 inhibitors for a period exceeding 3 months. Medication history of patients included was obtained from prescriptions in the electronic medical record. A total of 361 individuals were included in the SGLT2i group, with dapagliflozin being the most commonly used, administered to 257 subjects (71.2%), followed by empagliflozin in 56 subjects (15.5%), ertugliflozin in 29 subjects (8.0%), canagliflozin in 17 subjects (4.7%), and henagliflozin in 2 subjects (0.6%).

### Definitions and exposure measurements

The study involved interviews conducted by trained physicians, wherein subjects were queried regarding their smoking practices, alcohol intake, medical background, and the presence of cardiovascular disease and hypertension. Current smokers were those who consumed at least one cigarette per day for 12 months or longer, while current alcohol drinkers were defined as those drinking over 140 g of alcohol per week for 12 months or longer. Non-smokers were individuals who never smoked or who had quit smoking for more than 3 months. Non-alcohol drinkers were those who never drank alcohol or who had stopped drinking for the same duration.

### Outcome

The serum magnesium levels of hospitalized patients were obtained from their electronic medical records, and in cases where multiple measurements were taken during the admission period, the first recorded result was utilized. In accordance with our institution’s laboratory criteria, serum magnesium levels below 0.75 mmol/L were classified as hypomagnesemia.

### Covariates

To further investigate the potential of SGLT2 inhibitors (SGLT2i) as an independent predictor of hypomagnesemia, we conducted a logistic regression analysis, adjusting for a comprehensive set of confounding variables. These included age, gender, serum creatinine levels, alcohol consumption, smoking habits, hypertension status, cardiovascular disease, HbA1c levels, C-reactive protein levels, serum uric acid levels, alanine transaminase levels, and lipid profiles. Given the collinearity among lipid-related measurements, we included only triglycerides (TG) and low-density lipoprotein (LDL) cholesterol in our model. Additionally, we accounted for baseline medication use, encompassing antidiabetic agents such as insulins, metformin, thiazolidinediones, sulfonylureas, meglitinides, glucagon-like peptide-1 receptor agonists, alpha-glucosidase inhibitors, and DPP4 inhibitors. Body mass index (BMI) was calculated as weight (in kilograms) divided by height squared (in meters squared).

### Statistical analyses

The data are presented as means with standard deviations (mean ± SD) for quantitative variables, and as frequency counts with percentages (n, %) for categorical variables. Categorical characteristics among subjects were compared using the chi-square test (χ^2^), whereas continuous variables were analyzed with Student’s *t*-test.

To mitigate potential biases, propensity score matching (PSM) was conducted on all included covariates. In the 1:1 PSM, a nearest neighbor matching algorithm was employed to pair the SGLT2i group with the SGLT2i-free group, using a caliper width of 0.005. The factors included in the matching process were age, gender, body mass index (BMI), hypertension status, history of cardiovascular disease, current smoking status, current alcohol consumption, HbA1c levels, C-reactive protein levels, serum creatinine levels, serum uric acid levels, alanine transaminase levels, lipid profiles (including triglycerides (TG) and low-density lipoprotein (LDL)), and baseline medication use (antidiabetic medications such as insulins, metformin, thiazolidinediones, sulfonylureas, meglitinides, GLP1-RA, AGI, DPP-4i). These laboratory indicators are relatively simple and accessible, and can basically reflect the patient’s inflammatory, metabolic, and nutritional status.

**Figure 2:**
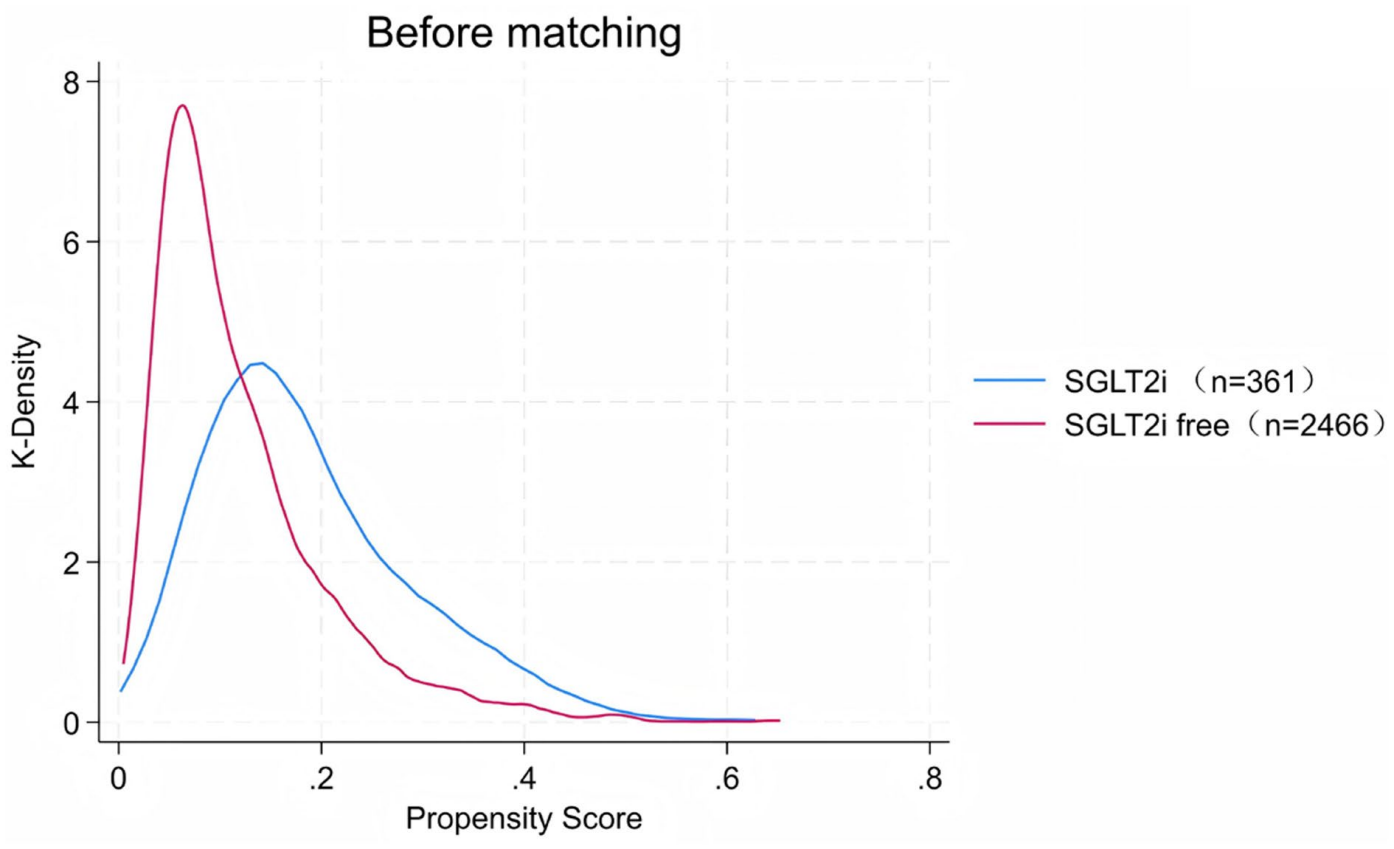
Distribution of propensity scores before matching SGLT2i.

**Figure 3:**
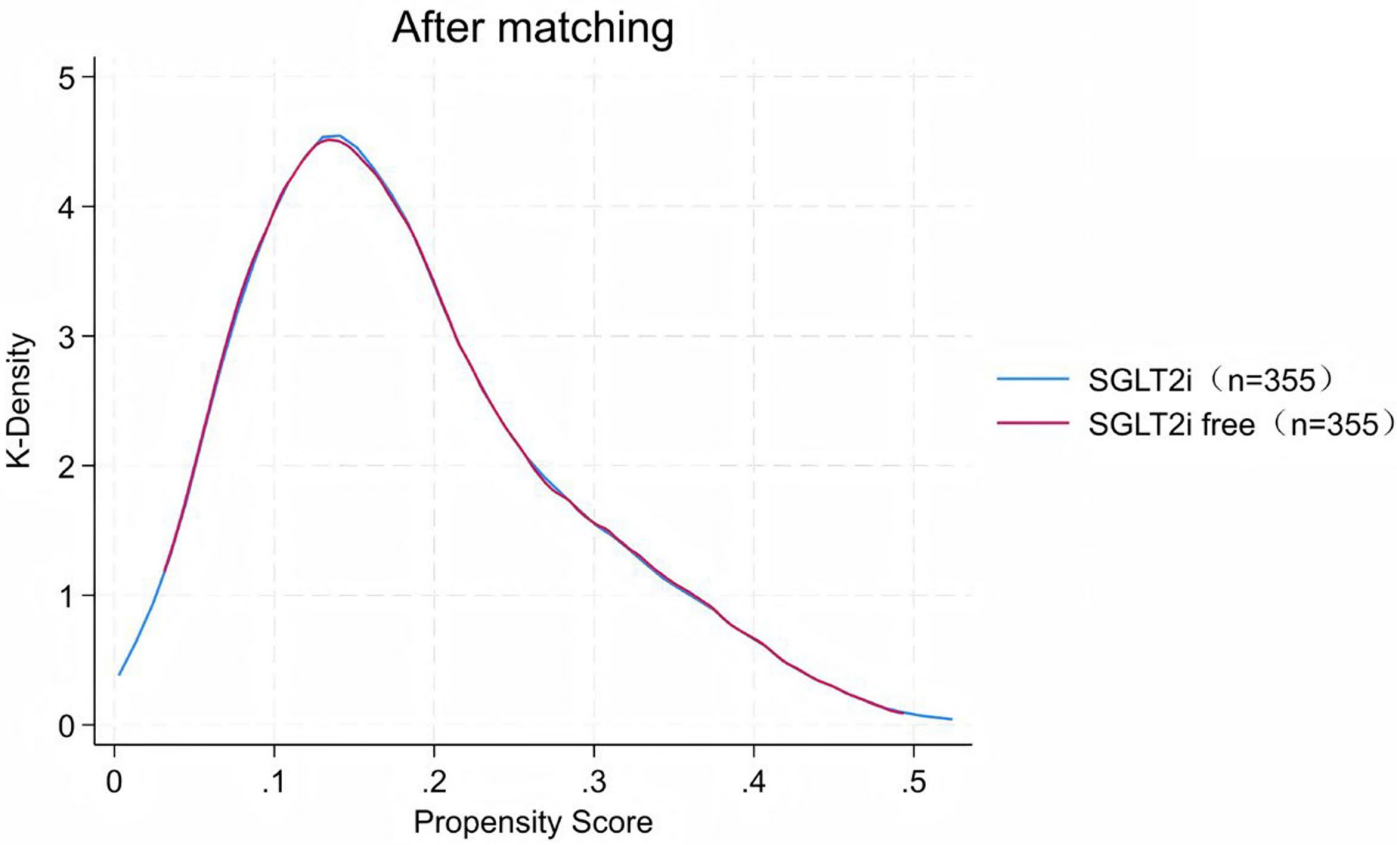
Distribution of propensity scores after matching SGLT2i.

Absolute standardized differences were calculated for all baseline characteristics to assess the balance between groups. A standardized mean difference (SMD) less than 0.1 was deemed indicative of balance and comparability. The distribution of propensity scores for patients pre- and post-propensity score matching (PSM) is illustrated in Figures 2, 3, respectively. Multivariate logistic regression analysis was employed to ascertain the odds ratios (ORs) and their associated 95% confidence intervals (CI). Statistical significance was pre-established at a two-sided *p*-value of less than 0.05. All statistical analyses were conducted using SPSS software version 27.0.

## Results

### Baseline characteristics of the study population before PSM

Table 1 provides a comprehensive overview of the baseline characteristics for both the SGLT2i group and the SGLT2i free group prior to propensity score matching analysis (PSM). A total of 2,827 subjects were enrolled in this study, with 361 belonging to the SGLT2i group and 2,466 assigned to the SGLT2i free group. Notably, 485 (17.16%) of the participants were diagnosed with Hypomagnesemia.

**Table 1:** Baseline clinical characteristics of the study participants according to SGLT2i before PSM.

Characters	SGLT2i (*n* = 361)	SGLT2i free (*n* = 2,466)	SMD
Demographic characteristics			
Age, years(±SD)	71.04 ± 5.97	72.82 ± 6.93	0.275
Gender, n(%)			
Female	162(44.9%)	1,145(46.4%)	0.030
Male	199(55.1%)	1,321(53.6%)	
BMI (kg/m^2^)	23.67 ± 3.02	23.64 ± 3.07	0.010
Current smoker, n(%)	83(23.0%)	526(21.3%)	0.041
Current alcohol drinker, n(%)	77(21.3%)	439(17.8%)	0.088
Co-morbidity			
Hypertension, n(%)	248(68.7%)	1,695(68.7%)	0.000
Cardiovascular disease, n(%)	61(16.9%)	304(12.3%)	
Co-medication			
AGI, n(%)	123(34.1%)	478(19.4%)	0.337
Metformin, n(%)	220(60.9%)	916(37.1%)	0.490
Thiazolidinediones, n(%)	3(0.8%)	29(1.2%)	0.040
Sulfonylureas (SU), n(%)	148(41.0%)	732(29.7%)	0.238
Glinides, n(%)	10(2.8%)	54(2.2%)	0.038
DPP-4i, n(%)	64(17.7%)	240(9.7%)	0.234
GLP1-RA, n(%)	9(2.5%)	18(0.7%)	0.144
Insulin, n(%)	93(25.8%)	382(15.5%)	0.257
Preoperative laboratory data			
HbA1c (%)	8.45 ± 1.81	8.14 ± 1.85	0.169
CRP (mg/L)	13.50 ± 35.20	15.90 ± 40.01	0.064
ALT (IU/L)	24.66 ± 28.23	26.26 ± 48.10	0.041
Albumin (g/L)	39.58 ± 3.66	38.73 ± 3.87	0.226
Serum creatinine (μmol/L)	70.11 ± 39.89	73.81 ± 66.40	0.068
Uric acid (μmol/L)	312.72 ± 95.56	319.95 ± 96.31	0.075
Triglyceride (mmol/L)	1.63 ± 1.05	1.69 ± 1.30	0.051
LDL-cholesterol (mmol/L)	2.46 ± 0.85	2.73 ± 0.94	0.301
Hypomagnesemia	35(9.7%)	450(18.2%)	
Serum magnesium (mmol/L)	0.841 ± 0.077	0.813 ± 0.083	

Compared to the SGLT2i-free cohort, the SGLT2i group demonstrated a significantly lower mean age and a higher prevalence of cardiovascular disease, which may be attributed to the use of SGLT2i as an anti-heart failure medication. The SGLT2i cohort also presented with a higher HbA1c level, indicating a greater propensity for the use of adjunctive hypoglycemic agents. Specifically, the utilization rates of metformin (60.9% vs. 37.1%, SMD = 0.490), sulfonylureas (41.0% vs. 29.7%, SMD = 0.238), DPP-4i(17.7% vs. 9.7%, SMD = 0.234), GLP1-RA (2.5% vs. 0.7%, SMD = 0.144), and insulin (25.8% vs. 15.5%, SMD = 0.257) were notably higher in combination therapy. Conversely, the SGLT2i group demonstrated a higher level of albumin (SMD = 0.226) and a lower level of LDL-cholesterol (SMD = 0.301).

### Baseline characteristics of the study participants after PSM

Considering that SGLT2i are associated with higher HbA1c levels and a greater propensity for the concomitant use of additional hypoglycemic agents, and given the observed differences in age, albumin levels, and triglyceride levels between the two groups, we employed propensity score matching (PSM) across all included covariates to mitigate potential biases.

Upon the implementation of the Propensity Score Matching (PSM) framework, we observed a substantial equilibrium in participant characteristics between the two groups, as detailed in Table 2. Notably, post-matching, no statistically significant differences were discernible in any of the variables between the groups, signifying a high level of comparability (SMD < 0.1). It is noteworthy that, even after the application of PSM, the SGLT2i group exhibited a persistently lower incidence of hypomagnesemia and higher serum magnesium levels.

**Table 2:** Baseline clinical characteristics of the study participants according to SGLT2i after PSM.

Characters	SGLT2i (*n* = 355)	SGLT2i free (*n* = 355)	SMD
Demographic characteristics			
Age, years(±SD)	71.05 ± 5.969	71.09 ± 5.765	0.007
Gender, n(%)			
Female	158(44.5%)	55(43.7%)	0.016
Male	197(55.5%)	200(56.3%)	
BMI (kg/m^2^)	23.68 ± 3.03	23.85 ± 3.13	0.055
Current smoker, n(%)	82(23.1%)	90(25.4%)	0.054
Current alcohol drinker, n(%)	76(21.4%)	82(23.1%)	0.041
Co-morbidity			
Hypertension, n(%)	245(69.0%)	247(69.6%)	0.013
Cardiovascular disease, n(%)	60(16.9%)	69(19.4%)	0.065
Co-medication			
AGI, n(%)	118(33.2%)	118(33.2%)	0.000
Metformin, n(%)	215(60.6%)	226(63.7%)	0.064
Thiazolidinediones, n(%)	3(0.8%)	2(0.6%)	0.024
Sulfonylureas (SU), n(%)	144(40.6%)	138(38.9%)	0.035
Glinides, n(%)	10(2.8%)	12(3.4%)	0.035
DPP-4i, n(%)	62(17.5%)	53(14.9%)	0.071
GLP1-RA, n(%)	8(2.3%)	6(1.7%)	0.043
Insulin, n(%)	91(25.6%)	86(24.2%)	0.032
Preoperative laboratory data			
HbA1c (%)	8.43 ± 1.79	8.32 ± 1.73	0.062
CRP (mg/L)	13.61 ± 35.33	13.56 ± 36.44	0.001
ALT (IU/L)	24.81 ± 28.42	26.66 ± 31.53	0.062
Albumin (g/L)	39.55 ± 3.67	39.41 ± 3.78	0.038
Serum creatinine (μmol/L)	70.28 ± 40.15	69.91 ± 31.63	0.010
Uric acid (μmol/L)	312.84 ± 95.90	313.78 ± 92.36	0.010
Triglyceride (mmol/L)	1.64 ± 1.05	1.63 ± 1.16	0.009
LDL-cholesterol (mmol/L)	2.48 ± 0.85	2.45 ± 0.82	0.036
Hypomagnesemia	35(9.9%)	84(23.7%)	
Serum magnesium (mmol/L)	0.841 ± 0.077	0.802 ± 0.077	

### Relationship between SGLT2i and hypomagnesemia

In both univariate and multivariate linear regression analyses, we observed a significant positive correlation between the utilization of SGLT2 inhibitors and serum magnesium levels, both before and after propensity score matching (Table 3).

**Table 3:** Correlation between SGLT2i and serum magnesium.

			Univariate analysis		Multivariate analysis	
	SGLT2	SGLT2 free	B(95% CI)	# *p*-value	B(95% CI)	# *p*-value
Outcomes before PSM						
Serum magnesium (mmol/L)	0.841 ± 0.077	0.813 ± 0.083	0.028 (0.019, 0.038)	*p* < 0.001*	0.036 (0.027, 0.045)	*p* < 0.001*
Outcomes after PSM						
Serum magnesium (mmol/L)	0.841 ± 0.077	0.802 ± 0.077	0.039 (0.027, 0.051)	*p* < 0.001*	0.038 (0.027, 0.050)	*p* < 0.001*

*indicates a significant difference, *p* < 0.05.

To further investigate the clinical significance, we defined hypomagnesemia as a serum Mg2 + concentration of less than 0.75 mmol/L based on the detection standards of our hospital’s laboratory. The results of the univariate and multivariate analyses are presented in Table 4 (before PSM) and Table 5 (after PSM). Prior to PSM, the overall prevalence of hypomagnesemia was 9.9% in the SGLT2i group and 23.7% in the SGLT2i-free group. Notably, the univariate analysis indicated a negative correlation between SGLT2i use and hypomagnesemia [0.481 (0.334–0.692), *p* < 0.001]. This trend persisted in the multivariate analysis [0.392 (0.267–0.576), *p* = 0.002]. Furthermore, we observed that the use of metformin and thiazolidinediones was associated with an increased incidence of hypomagnesemia. Additionally, elevated levels of HbA1c and uric acid were also correlated with an augmented risk of hypomagnesemia, suggesting potentially poorer metabolic homeostasis. Conversely, elevated levels of albumin and LDL-cholesterol were negatively correlated with the incidence of hypomagnesemia, potentially indicating better nutritional status and thus a higher intake of dietary magnesium. These findings were consistent across both univariate and multivariate analyses.

**Table 4:** Univariate and multivariate analysis for possible associated factors of hypomagnesemia before PSM.

Variables	Univariate analysis	# *p*-value	Multivariate analysis	# *p*-value
	OR(95% CI)		OR(95% CI)	
Demographic characteristics				
Age (years)	1.014(1.000–1.028)	0.055	0.993(0.978–1.009)	0.394
Gender, female	1.008(0.828–1.226)	0.938	1.269(0.985–1.635)	0.066
BMI (kg/m^2^)	0.972(0.941–1.004)	0.084	0.967(0.934–1.001)	0.057
Current smoker	1.083(0.857–1.370)	0.503	1.047(0.769–1.424)	0.772
Current alcohol drinker	1.076(0.839–1.381)	0.563	1.126(0.825–1.538)	0.455
Co-morbidity				
Hypertension	1.147(0.926–1.422)	0.210	1.247(0.987–1.574)	0.064
Cardiovascular disease	1.053(0.790–1.405)	0.723	1.234(0.906–1.681)	0.183
Co-medication				
SGLT2i	0.481(0.334–0.692)	*p* < 0.001*	0.392(0.267–0.576)	*p* < 0.001*
AGI	1.059(0.836–1.341)	0.635	0.882(0.678–1.148)	0.350
Metformin	1.662(1.366–2.024)	*p* < 0.001*	2.194(1.744–2.761)	*p* < 0.001*
Thiazolidinediones	2.565(1.228–5.355)	0.009*	4.470(1.978–10.102)	*p* < 0.001*
Sulfonylureas (SU)	0.977(0.791–1.208)	0.832	0.785(0.615–1.003)	0.053
Glinides	0.685(0.324–1.446)	0.318	0.651(0.298–1.422)	0.282
DPP-4i	1.048(0.768–1.432)	0.766	1.006(0.717–1.412)	0.971
GLP1-RA	0.838(0.289–2.435)	0.746	0.882(0.292–2.662)	0.823
Insulin	1.384(1.083–1.769)	0.009*	1.181(0.895–1.557)	0.239
Preoperative laboratory data				
HbA1c (%)	1.099(1.046–1.155)	*p* < 0.001*	1.096(1.036–1.160)	0.001*
CRP (mg/L)	1.004(1.002–1.006)	*p* < 0.001*	1.002(0.999–1.004)	0.224
ALT (IU/L)	0.999(0.997–1.002)	0.662	1.000(0.997–1.002)	0.844
Albumin (g/L)	0.901(0.878–0.923)	*p* < 0.001*	0.895(0.867–0.923)	*p* < 0.001*
Serum creatinine (μmol/L)	1.000(0.998–1.001)	0.620	0.996(0.993–0.999)	0.004*
Uric acid (μmol/L)	1.002(1.001–1.003)	*p* < 0.001*	1.004(1.003–1.005)	*p* < 0.001*
Triglyceride (mmol/L)	0.919(0.833–1.014)	0.092	0.947(0.850–1.055)	0.324
LDL-cholesterol (mmol/L)	0.823(0.737–0.919)	*p* < 0.001*	0.814(0.718–0.922)	0.001*

The bold values and * both indicate a significant difference, *p* < 0.05.

**Table 5:** Univariate and multivariate analysis for possible associated factors of hypomagnesemia after PSM.

Variables	Univariate analysis	# *p*-value	Multivariate analysis	# *p*-value
	OR(95% CI)		OR(95% CI)	
Demographic characteristics				
Age (years)	1.007(0.974–1.041)	0.694	0.998(0.961–1.036)	0.907
Gender, female	0.733(0.489–1.098)	0.131	0.689(0.406–1.17)	0.168
BMI (kg/m^2^)	0.991(0.929–1.056)	0.772	0.980(0.912–1.052)	0.571
Current smoker	1.066(0.676–1.680)	0.784	0.782(0.431–1.419)	0.419
Current alcohol drinker	1.091(0.685–1.739)	0.714	1.042(0.57–1.903)	0.894
Co-morbidity				
Hypertension	1.459(0.928–2.295)	0.101	1.723(1.047–2.834)	0.032*
Cardiovascular disease	0.893(0.529–1.508)	0.673	0.780(0.442–1.379)	0.393
Co-medication				
SGLT2i	0.353(0.230–0.540)	*p* < 0.001*	0.325(0.208–0.506)	*p* < 0.001*
AGI	1.020(0.672–1.549)	0.924	0.899(0.566–1.429)	0.653
Metformin	1.820(1.175–2.819)	0.007*	2.022(1.239–3.301)	0.005*
Thiazolidinediones	3.350(0.554–20.272)	0.163	6.301(0.924–42.968)	0.060
Sulfonylureas (SU)	1.076(0.721–1.605)	0.722	0.940(0.596–1.481)	0.789
Glinides	0.779(0.227–2.674)	0.690	0.723(0.195–2.677)	0.627
DPP-4i	1.133(0.673–1.906)	0.638	1.195(0.680–2.100)	0.536
GLP1-RA	0.825(0.182–3.734)	0.802	1.091(0.227–5.233)	0.914
Insulin	1.384(0.896–2.138)	0.141	1.188(0.726–1.945)	0.493
Preoperative laboratory data				
HbA1c (%)	1.117(1.005–1.241)	0.040*	1.141(1.011–1.287)	0.033*
CRP (mg/L)	1.000(0.994–1.005)	0.893	1.001(0.994–1.007)	0.833
ALT (IU/L)	1.000(0.993–1.007)	0.995	1.001(0.993–1.008)	0.889
Albumin (g/L)	0.969(0.920–1.022)	0.246	0.966(0.907–1.029)	0.288
Serum creatinine (μmol/L)	1.000(0.995–1.006)	0.881	0.997(0.989–1.004)	0.382
Uric acid (μmol/L)	1.002(1.000–1.004)	0.123	1.003(1.000–1.005)	0.035*
Triglyceride (mmol/L)	0.840(0.673–1.048)	0.122	0.846(0.654–1.096)	0.206
LDL-cholesterol (mmol/L)	0.843(0.661–1.075)	0.168	0.853(0.644–1.129)	0.266

The bold values and * both indicate a significant difference, *p* < 0.05.

Upon employing the PSM methodology, the robust negative correlation between SGLT2i and the incidence of hypomagnesemia persisted in both univariate [0.353 (95% CI: 0.230–0.540), *p* < 0.001] and multivariate analyses [0.325 (95% CI: 0.208–0.506), *p* < 0.001]. The use of metformin was associated with a positive correlation to the occurrence of hypomagnesemia in both univariate and multivariate analyses, aligning with pre-PSM results. Conversely, the correlation with thiazolidinediones lost significance post-PSM. Furthermore, elevated HbA1c levels were indicative of a higher incidence of hypomagnesemia subsequent to PSM, corroborating pre-PSM observations. Post-PSM, uric acid levels did not show a correlation with hypomagnesemia in univariate analysis; however, in multivariate analysis, higher uric acid levels were found to have a positive correlation with hypomagnesemia, which corroborates pre-PSM outcomes.

Considering that renal function (eGFR staging) and continuous creatinine as covariates may lead to inconsistent results, we compared the baseline data of serum creatinine levels, eGFR levels, and eGFR categories before and after PSM, and the results showed that all groups were comparable. In addition, we included the above three as confounding variables in the analysis, respectively. Both before and after PSM, there was a significant negative correlation between SGLT2i and the incidence of hypomagnesemia (Supplementary Table 1.2).

### Subgroup analysis

**Figure 4:**
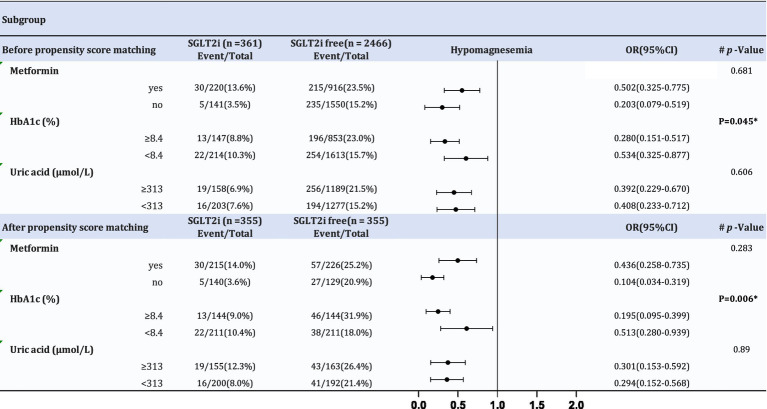
Subgroup analysis before and after matching.

Upon comprehensive covariate adjustment, Figure 4 presents the compelling outcomes of a subgroup analysis. We stratified patients based on post-PSM average levels of HbA1c and uric acid, using 8.4% (the post-PSM mean HbA1c) as the threshold for HbA1c and 313 μmol/L (the post-PSM mean uric acid) for uric acid levels. Notably, HbA1c levels appear to modulate the inverse relationship between SGLT2i and the incidence of hypomagnesemia, both before and after PSM. In patients with T2DM and HbA1c levels of 8.4% or greater, the negative correlation between SGLT2i and the incidence of hypomagnesemia is more significant compared to those with HbA1c levels below 8.4%.

## Discussion

It has been reported that hypomagnesemia, characterized by low serum magnesium levels, affects 13.5–47.7% of non-hospitalized patients with type 2 diabetes, compared to a prevalence of 2.5–15% in non-diabetic individuals. The prevailing belief is that diabetes itself may precipitate hypomagnesemia (19, 20). Numerous studies have identified a negative correlation between blood glucose control and serum magnesium levels (21, 22), a finding that aligns with our research, indicating that higher HbA1c levels in T2DM patients are associated with an increased risk of hypomagnesemia. However, the causal relationship between these factors remains unclear. Some research suggests that hypomagnesemia may increase the risk of developing type 2 diabetes (23). The efficacy of oral magnesium supplementation in improving glycemic control and insulin resistance is still a matter of debate. Evidence suggests that oral magnesium supplementation can reduce insulin resistance and C-peptide concentrations, and enhance glycemic control indicators in T2DM patients (24, 25). Conversely, other studies indicate that magnesium supplementation has no effect on glycemic control or plasma lipid concentrations (26–28).

Extensive research has indicated that hypomagnesemia is associated with both microvascular and macrovascular complications of diabetes. Hypomagnesemia has been shown to increase the risk of conditions such as coronary heart disease (29, 30), diabetic retinopathy (31), and diabetic foot syndrome (32, 33). Studies have also found that low serum magnesium levels can lead to deterioration in renal function (34). A recent retrospective study revealed that hypomagnesemia is independently associated with a higher risk of albuminuria (35). Our current study has found a positive correlation between low serum albumin and hypomagnesemia, which may be due to the promotion of urinary albumin excretion by hypomagnesemia, resulting in decreased serum albumin levels.

Magnesium is filtered at the glomerulus and reabsorbed at various tubular sites via a passive paracellular pathway, relying on positive lumen electrical potential generated by the NKCC (Na - K - 2Cl) transporter and renal outer medullary K channel (ROMK) (36). Most reabsorption occurs in the thick ascending limb via claudin −16/19 tight junctions (37). NKCC/ROMK-generated positive lumen potential drives magnesium uptake. SGLT2i block SGLT2 in the proximal renal tubule, leading to increased urinary glucose and sodium excretion, thereby reducing blood glucose levels and serum sodium concentrations (38), SGLT2 inhibition increases distal sodium delivery, enhancing NKCC/NCC(Na-Cl) activity to further raise lumen potential and promote magnesium reabsorption.

In the distal tubule, Mg2 + is reabsorbed from the pre-urine via TRPM6 channels. It is widely accepted that the inactivation of TRPM6 channels is associated with insulin resistance, and SGLT2i can improve insulin sensitivity (39, 40). Furthermore, TRPM6 channel activity is regulated by epidermal growth factor (EGF) (41). A recent study measuring EGF levels in the kidneys of T2DM patients treated with SGLT2i found that SGLT2i increases EGF expression in these patients (42).

SGLT2 inhibitors induce a state akin to fasting in the body, characterized by enhanced gluconeogenesis and ketogenesis, a feature not shared by other antidiabetic agents (43). This may account for the observed outcomes, as the SGLT2i group exhibited lower levels of Triglyceride and LDL-cholesterol.

The observed negative correlation between the antidiabetic drug metformin and plasma Mg levels may be attributed to metformin-induced diarrhea (5, 8). The primary mechanism responsible for the efflux of Mg2 + from cells is the Na+/Mg2 + exchanger (NME), which is regulated (activated) through phosphorylation mediated by cAMP-dependent protein kinase A (PKA) (44, 45). Metformin activates the cAMP/PKA pathway, which may be associated with the hypomagnesemia observed in previous studies and is consistent with our current findings.

Our findings suggest that in the context of managing T2DM among elderly patients, it may be prudent to consider serum magnesium levels when determining the appropriate glucose - lowering medication to initiate. SGLT2i are associated with a lower incidence of hypomagnesemia, particularly in the geriatric population with T2DM. However, this study has several limitations: significant baseline differences (HbA1c, metformin/insulin use, cardiovascular disease prevalence) persisted despite PSM, with potential residual confounding and indication bias. Furthermore, this single—center coastal study, whose participants are influenced by regional and dietary factors, lacks dietary data—a critical limitation. In particular, seafood (including fish, crustaceans, and mollusks), a food group known to be rich in magnesium (46, 47), may affect the serum magnesium levels of the population due to participants’ dietary preferences for such foods. The SGLT2i group has higher albumin and LDL levels, which means this experimental group has a better nutritional status. A better nutritional status often implies a higher serum magnesium level. To minimize this influence, we used PSM to reduce interference. Additionally, as a cross - sectional retrospective study lacking longitudinal follow - up data, further pre/post - SGLT2i serum magnesium comparisons are needed to validate the hypothesis. First, hypomagnesemia - SGLT2i associations and variable interactions require validation via rigorous prospective studies. Second, undifferentiated SGLT2i agents may confound results. Finally, limited data necessitates larger, diverse datasets to confirm findings.

## Conclusion

There is a significant negative correlation between SGLT2i and the incidence of hypomagnesemia in T2DM patients over 60. In those with higher HbA1c levels (≥8.4%), the negative correlation between SGLT2i and hypomagnesemia is more significant. When considering the prescription of hypoglycemic agents for T2D patients with hypomagnesemia, our study may provide valuable insights for the decision—making process.

## Data Availability

The raw data supporting the conclusions of this article will be made available by the authors, without undue reservation.
