# Supplementary material for: Sodium-glucose co-transporter 2 inhibitors are negatively correlated with hypomagnesemia in elderly patients with type 2 diabetes
Source: Front Nutr. 2026 May 14;13:1790168. doi: 10.3389/fnut.2026.1790168 (PMC13219964; doi:10.3389/fnut.2026.1790168)

| **Supplementary Table 1：Baseline clinical characteristics of the study participants according to SGLT2i before PSM** | | | |
| --- | --- | --- | --- |
| **before PSM** | **SGLT2i**  **(n =361)** | **SGLT2i free**  **(n = 2466)** | **SMD** |
| **Serum creatinine (μmol/L)** | 70.11±39.89 | 73.81±66.40 | 0.068 |
| **eGFR(ml/min/1.73m²)** | 87.44±18.00 | 85.74±18.51 | 0.093 |
| **eGFR categories** |  |  |  |
| **G1** | 244(67.6%) | 1571(63.7%) | 0.082 |
| **G2** | 89(24.7%) | 696(28.2%) | 0.079 |
| **G3a** | 17(4.7%) | 99(4.0%) | 0.034 |
| **G3b** | 7(1.9%) | 49(2.0%) | 0.007 |
| **G4** | 2(0.6%) | 31(1.3%) | 0.072 |
| **G5** | 2(0.6%) | 20(0.8%) | 0.035 |
| **after PSM** | **SGLT2i**  **(n = 355)** | **SGLT2i free**  **(n = 355)** | **SMD** |
| **Serum creatinine (μmol/L)** | 70.28±40.15 | 69.91±31.63 | 0.010 |
| **eGFR(ml/min/1.73m²)** | 81.20±20.11 | 80.76±19.38 | 0.022 |
| **eGFR categories** |  |  |  |
| **G1** | 160(45.1%) | 148(41.7%) | 0.069 |
| **G2** | 140(39.4%) | 696(43.1%) | 0.075 |
| **G3a** | 29(8.2%) | 33(9.3%) | 0.039 |
| **G3b** | 22(6.2%) | 14(3.9%) | 0.105 |
| **G4** | 2(0.6%) | 6(1.7%) | 0.103 |
| **G5** | 2(0.6%) | 1(0.3%) | 0.045 |

**Supplementary Table 1：. * indicates a significant difference, p < 0.05. SGLT2i, sodium-glucose cotransporter-2 inhibitor; PSM, propensity score matching; SMD, standardized mean difference；eGFR: Calculated using the CKD - EPI Cr formula;**

| **Supplementary Table 2：Univariate and multivariate analysis for possible associated factors of hypomagnesemia** | | | | |
| --- | --- | --- | --- | --- |
|  | **Univariate analysis** |  | **Multivariate analysis** |  |
| **before PSM** | **OR(95% CI)** | **# *p*-Value** | **OR(95% CI)** | **# *p*-Value** |
| **Serum creatinine (μmol/L)^a^** | 1.000(0.998-1.001) | P=0.620 | 0.996(0.993-0.999) | **P=0.004*** |
| **eGFR(ml/min/1.73m²)^b^** | 0.996(0.991-1.001) | P=0.138 | 1.009(1.002-1.016) | P=0.008 |
| **eGFR categories ^c^** | 1.032(0.919-1.159) | P=0.589 | 0.774(0.669-0.897) | **P=0.001*** |
|  |  |  |  |  |
| **^a^SGLT2i** | 0.481(0.334-0.692) | **P<0.001*** | 0.392(0.267-0.576) | **P<0.001*** |
| **^b^SGLT2i** |  |  | 0.391(0.266-0.574) | **P<0.001*** |
| **^c^SGLT2i** |  |  | 0.393(0.267-0.578) | **P<0.001*** |
| **after PSM** | **OR(95% CI)** | **# *p*-Value** | **OR(95% CI)** | **# *p*-Value** |
| **Serum creatinine (μmol/L)^a^** | 1.000(0.995-1.006) | P=0.881 | 0.997(0.989-1.004) | P=0.382 |
| **eGFR(ml/min/1.73m²)^b^** | 0.994(0.985-1.004) | P=0.231 | 0.999(0.985-1.012) | P=0.827 |
| **eGFR categories ^c^** | 1.145(0.931-1.408) | P=0.199 | 1.043(0.786-1.385) | P=0.769 |
| **^a^SGLT2i** | 0.353(0.230-0.540) | **P<0.001*** | 0.325(0.208-0.506) | **P<0.001*** |
| **^b^SGLT2i** |  |  | 0.326(0.209-0.508) | **P<0.001*** |
| **^c^SGLT2i** |  |  | 0.326(0.209-0.508) | **P<0.001*** |

**Supplementary Table 2：* indicates a significant difference (p < 0.05). SGLT2i: sodium-glucose cotransporter-2 inhibitor; PSM: propensity score matching; SMD: standardized mean difference; eGFR: calculated using the CKD-EPI Cr formula. All covariates were included in the multivariate analysis, except that serum creatinine was replaced by eGFR or eGFR categories respectively. eGFR categories (G1, G2, G3a, G3b, G4/5 [combined into one group due to insufficient data]) were treated as continuous categorical variables. a Multivariate analysis of SGLT2i included all covariates including serum creatinine; b Multivariate analysis of SGLT2i included all covariates including eGFR; c Multivariate analysis of SGLT2i included all covariates including eGFR categories.**

**Supplementary figure 1**


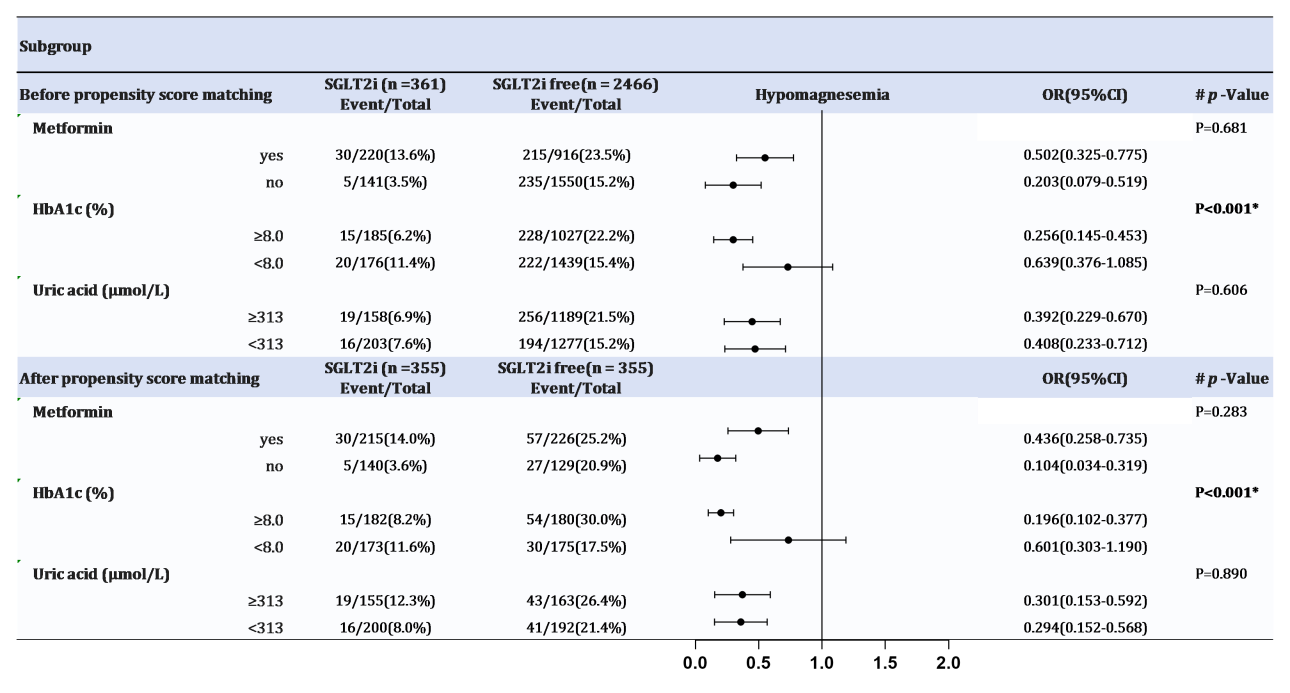

Supplement: Supplementary file 1 [file Table_1.docx]
